# (2*E*)-1-(4,4′′-Difluoro-5′-meth­oxy-1,1′:3′,1′′-terphenyl-4′-yl)-3-(2-fluoro­phen­yl)prop-2-en-1-one

**DOI:** 10.1107/S1600536812024981

**Published:** 2012-06-13

**Authors:** Hoong-Kun Fun, Wan-Sin Loh, S. Samshuddin, B. Narayana, B. K. Sarojini

**Affiliations:** a X-ray Crystallography Unit, School of Physics, Universiti Sains Malaysia, 11800 USM, Penang, Malaysia; b Department of Studies in Chemistry, Mangalore University, Mangalagangotri 574 199, India; c Department of Chemistry, P.A. College of Engineering, Nadupadavu, Mangalore 574 153, India

## Abstract

In the title compound, C_28_H_19_F_3_O_2_, the central benzene ring forms dihedral angles of 48.69 (6), 60.93 (6) and 42.06 (6)° with the fluoro­benzene rings. In the crystal, inter­molecular C—H⋯O and C—H⋯F hydrogen bonds link the mol­ecules, forming an undulating two-dimensional network parallel to the *bc* plane. C—H⋯π inter­actions further consolidate the crystal packing.

## Related literature

For background to terphenyl chalcones, see: Fun *et al.* (2011 ▶); Fun, Hemamalini *et al.* (2012 ▶). For a related structure, see: Fun, Loh *et al.* (2012 ▶). For the stability of the temperature controller used in the data collection, see: Cosier & Glazer (1986 ▶).
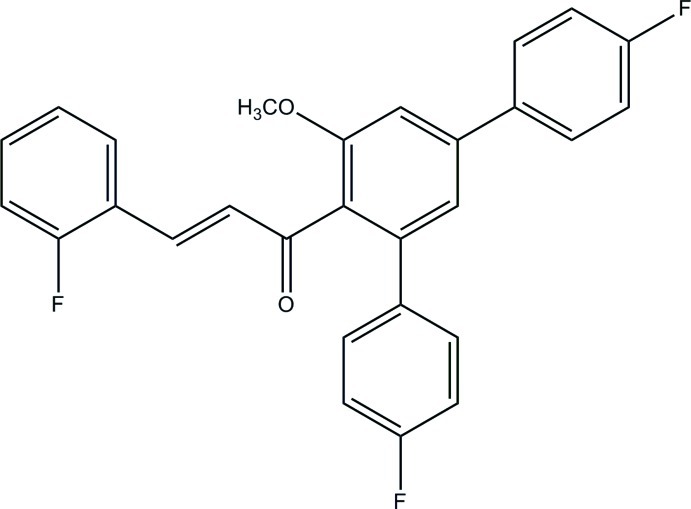


## Experimental

### 

#### Crystal data

C_28_H_19_F_3_O_2_
*M* *_r_* = 444.43
Monoclinic, 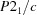

*a* = 13.7592 (1) Å
*b* = 6.7898 (1) Å
*c* = 22.4361 (3) Å
β = 101.908 (1)°
*V* = 2050.92 (4) Å^3^
*Z* = 4
Mo *K*α radiation
μ = 0.11 mm^−1^
*T* = 100 K
0.32 × 0.24 × 0.12 mm

#### Data collection

Bruker SMART APEXII CCD area-detector diffractometer
Absorption correction: multi-scan (*SADABS*; Bruker, 2009 ▶) *T*_min_ = 0.966, *T*_max_ = 0.987
28707 measured reflections
7478 independent reflections
5317 reflections with *I* > 2σ(*I*)
*R*_int_ = 0.041

#### Refinement

*R*[*F* ^2^ > 2σ(*F* ^2^)] = 0.058
*wR*(*F* ^2^) = 0.140
*S* = 1.03
7478 reflections
299 parameters
H-atom parameters constrained
Δρ_max_ = 0.53 e Å^−3^
Δρ_min_ = −0.26 e Å^−3^

### 

Data collection: *APEX2* (Bruker, 2009 ▶); cell refinement: *SAINT* (Bruker, 2009 ▶); data reduction: *SAINT*; program(s) used to solve structure: *SHELXTL* (Sheldrick, 2008 ▶); program(s) used to refine structure: *SHELXTL*; molecular graphics: *SHELXTL*; software used to prepare material for publication: *SHELXTL* and *PLATON* (Spek, 2009 ▶).

## Supplementary Material

Crystal structure: contains datablock(s) global, I. DOI: 10.1107/S1600536812024981/is5146sup1.cif

Structure factors: contains datablock(s) I. DOI: 10.1107/S1600536812024981/is5146Isup2.hkl

Supplementary material file. DOI: 10.1107/S1600536812024981/is5146Isup3.cml

Additional supplementary materials:  crystallographic information; 3D view; checkCIF report

## Figures and Tables

**Table 1:** Hydrogen-bond geometry (Å, °) *Cg*1 is the centroid of the C7–C12 ring.

*D*—H⋯*A*	*D*—H	H⋯*A*	*D*⋯*A*	*D*—H⋯*A*
C4—H4*A*⋯O2^i^	0.95	2.40	3.3008 (18)	158
C19—H19*A*⋯F2^ii^	0.95	2.54	3.2326 (18)	130
C24—H24*A*⋯*Cg*1^iii^	0.95	2.84	3.4579 (15)	124
C28—H28*C*⋯*Cg*1^iv^	0.98	2.86	3.5461 (16)	128

Symmetry codes: (i) 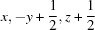; (ii) 
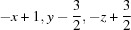
; (iii) 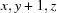; (iv) 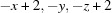.

**Fig. 1.**
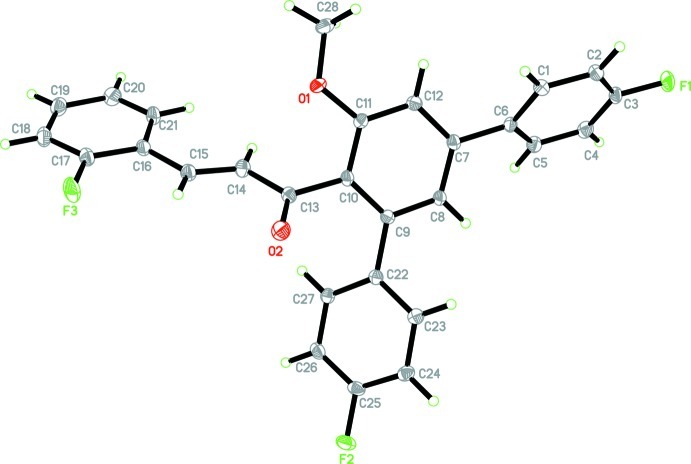
The molecular structure of the title compound, showing 50% probability displacement ellipsoids.

**Fig. 2.**
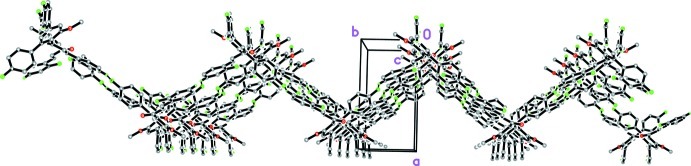
The crystal packing of the title compound, viewed along the c axis, showing the undulating two-dimensional network parallel to the bc plane. H atoms not involved in the intermolecular interactions (dashed lines) have been omitted for clarity.

## supplementary crystallographic information

### Comment

In continuation of our work on synthesis of terphenyl chalcones (Fun *et al.*, 2011), the title compound is prepared and its crystal structure is reported. The starting material of the title compound was prepared from 4,4'-difluoro chalcone by several steps (Fun, Hemamalini *et al.*, 2012).

In the title compound (Fig. 1), the central benzene ring (C7–C12) forms dihedral angles of 48.69 (6), 60.93 (6) and 42.06 (6)°, respectively, with the fluorobenzene rings C1–C6/F1, C16–C21/F3 and C22–C27/F2. Bond lengths and angles are within the normal ranges and are comparable with the related structure (Fun, Loh *et al.*, 2012).

In the crystal packing (Fig. 2), intermolecular C4—H4A···O2 and C19—H19A···F2 hydrogen bonds (Table 1) link the molecules to form undulating two-dimensional network parallel to the *bc* plane. C—H···π interactions (Table 1), involving the central benzene ring, further consolidate the crystal packing.

### Experimental

To a mixture of 1-(4,4''-difluoro-5'-methoxy-1,1':3',1''-terphenyl-4'-yl) ethanone (0.338 g, 0.001 mol) and 2-fluorobenzaldehyde (0.124 g, 0.001 mol) in 30 ml ethanol, 0.5 ml of 10% sodium hydroxide solution was added and stirred at 5–10°C for 3 h. The precipitate formed was collected by filtration and purified by recrystallization from ethanol. The single-crystal was grown from methanol by slow evaporation method and yield of the compound was 74%. *M.p.*: 453 K.

### Refinement

All the H atoms were positioned geometrically and were refined with a riding model with *U*_iso_(H) = 1.2 or 1.5 *U*_eq_(C) (C—H = 0.95 or 0.98 Å). A rotating group model was applied to the methyl group. In the final refinement, one outliner (-13 1 30) was omitted.

### Crystal data

**Table:**

C_28_H_19_F_3_O_2_	*F*(000) = 920
*M**_r_* = 444.43	*D*_x_ = 1.439 Mg m^−^^3^
Monoclinic, *P*2_1_/*c*	Mo *K*α radiation, λ = 0.71073 Å
Hall symbol: -P 2ybc	Cell parameters from 6446 reflections
*a* = 13.7592 (1) Å	θ = 3.1–32.6°
*b* = 6.7898 (1) Å	µ = 0.11 mm^−^^1^
*c* = 22.4361 (3) Å	*T* = 100 K
β = 101.908 (1)°	Block, yellow
*V* = 2050.92 (4) Å^3^	0.32 × 0.24 × 0.12 mm
*Z* = 4	

### Data collection

**Table:**

Bruker SMART APEXII CCD area-detector diffractometer	7478 independent reflections
Radiation source: fine-focus sealed tube	5317 reflections with *I* > 2σ(*I*)
Graphite monochromator	*R*_int_ = 0.041
φ and ω scans	θ_max_ = 32.7°, θ_min_ = 1.9°
Absorption correction: multi-scan (*SADABS*; Bruker, 2009)	*h* = −20→20
*T*_min_ = 0.966, *T*_max_ = 0.987	*k* = −10→10
28707 measured reflections	*l* = −34→31

### Refinement

**Table:**

Refinement on *F*^2^	Primary atom site location: structure-invariant direct methods
Least-squares matrix: full	Secondary atom site location: difference Fourier map
*R*[*F*^2^ > 2σ(*F*^2^)] = 0.058	Hydrogen site location: inferred from neighbouring sites
*wR*(*F*^2^) = 0.140	H-atom parameters constrained
*S* = 1.03	*w* = 1/[σ^2^(*F*_o_^2^) + (0.0578*P*)^2^ + 0.9821*P*] where *P* = (*F*_o_^2^ + 2*F*_c_^2^)/3
7478 reflections	(Δ/σ)_max_ = 0.001
299 parameters	Δρ_max_ = 0.53 e Å^−^^3^
0 restraints	Δρ_min_ = −0.26 e Å^−^^3^

### Special details

**Table:**

Experimental. The crystal was placed in the cold stream of an Oxford Cryosystems Cobra open-flow nitrogen cryostat (Cosier & Glazer, 1986) operating at 100.0 (1) K.
Geometry. All e.s.d.'s (except the e.s.d. in the dihedral angle between two l.s. planes) are estimated using the full covariance matrix. The cell e.s.d.'s are taken into account individually in the estimation of e.s.d.'s in distances, angles and torsion angles; correlations between e.s.d.'s in cell parameters are only used when they are defined by crystal symmetry. An approximate (isotropic) treatment of cell e.s.d.'s is used for estimating e.s.d.'s involving l.s. planes.
Refinement. Refinement of *F*^2^ against ALL reflections. The weighted *R*-factor *wR* and goodness of fit *S* are based on *F*^2^, conventional *R*-factors *R* are based on *F*, with *F* set to zero for negative *F*^2^. The threshold expression of *F*^2^ > σ(*F*^2^) is used only for calculating *R*-factors(gt) *etc*. and is not relevant to the choice of reflections for refinement. *R*-factors based on *F*^2^ are statistically about twice as large as those based on *F*, and *R*- factors based on ALL data will be even larger.

### Fractional atomic coordinates and isotropic or equivalent isotropic displacement parameters (Å^2^)

**Table:**

	*x*	*y*	*z*	*U*_iso_*/*U*_eq_	
F1	1.03222 (7)	0.04726 (14)	1.35190 (4)	0.0244 (2)	
F2	0.49534 (7)	0.88570 (14)	0.94449 (4)	0.0250 (2)	
F3	0.66995 (7)	0.00341 (15)	0.68457 (4)	0.0257 (2)	
O1	0.88761 (8)	−0.12968 (16)	0.95428 (5)	0.0185 (2)	
O2	0.77791 (8)	0.32186 (16)	0.88556 (5)	0.0206 (2)	
C1	1.01130 (10)	0.0527 (2)	1.18851 (7)	0.0159 (3)	
H1A	1.0529	0.0473	1.1596	0.019*	
C2	1.05342 (11)	0.0473 (2)	1.25032 (7)	0.0173 (3)	
H2A	1.1234	0.0407	1.2642	0.021*	
C3	0.99095 (11)	0.0519 (2)	1.29097 (6)	0.0169 (3)	
C4	0.88906 (11)	0.0613 (2)	1.27336 (7)	0.0183 (3)	
H4A	0.8481	0.0613	1.3026	0.022*	
C5	0.84821 (10)	0.0706 (2)	1.21143 (7)	0.0167 (3)	
H5A	0.7782	0.0804	1.1982	0.020*	
C6	0.90827 (10)	0.0659 (2)	1.16825 (6)	0.0138 (3)	
C7	0.86489 (10)	0.0826 (2)	1.10200 (6)	0.0134 (2)	
C8	0.79555 (9)	0.2298 (2)	1.08064 (6)	0.0131 (2)	
H8A	0.7720	0.3111	1.1091	0.016*	
C9	0.76002 (9)	0.2601 (2)	1.01833 (6)	0.0124 (2)	
C10	0.79304 (9)	0.1352 (2)	0.97656 (6)	0.0125 (2)	
C11	0.85927 (9)	−0.0180 (2)	0.99851 (6)	0.0131 (2)	
C12	0.89648 (10)	−0.0432 (2)	1.06048 (6)	0.0141 (3)	
H12A	0.9430	−0.1450	1.0745	0.017*	
C13	0.76484 (9)	0.1637 (2)	0.90865 (6)	0.0140 (3)	
C14	0.72077 (10)	−0.0086 (2)	0.87229 (6)	0.0167 (3)	
H14A	0.7023	−0.1201	0.8930	0.020*	
C15	0.70598 (10)	−0.0131 (2)	0.81141 (7)	0.0164 (3)	
H15A	0.7247	0.0998	0.7914	0.020*	
C16	0.66305 (10)	−0.1795 (2)	0.77340 (6)	0.0157 (3)	
C17	0.64452 (10)	−0.1661 (2)	0.71015 (7)	0.0174 (3)	
C18	0.60072 (11)	−0.3144 (2)	0.67147 (7)	0.0206 (3)	
H18A	0.5895	−0.2983	0.6285	0.025*	
C19	0.57362 (11)	−0.4870 (2)	0.69676 (7)	0.0212 (3)	
H19A	0.5423	−0.5899	0.6711	0.025*	
C20	0.59242 (11)	−0.5092 (2)	0.75979 (7)	0.0211 (3)	
H20A	0.5748	−0.6281	0.7772	0.025*	
C21	0.63683 (11)	−0.3579 (2)	0.79707 (7)	0.0188 (3)	
H21A	0.6499	−0.3758	0.8400	0.023*	
C22	0.68836 (9)	0.4236 (2)	0.99834 (6)	0.0125 (2)	
C23	0.70360 (10)	0.6066 (2)	1.02758 (6)	0.0147 (3)	
H23A	0.7592	0.6239	1.0602	0.018*	
C24	0.63912 (10)	0.7636 (2)	1.00989 (7)	0.0160 (3)	
H24A	0.6503	0.8879	1.0296	0.019*	
C25	0.55861 (10)	0.7334 (2)	0.96299 (7)	0.0167 (3)	
C26	0.53926 (10)	0.5546 (2)	0.93351 (6)	0.0163 (3)	
H26A	0.4827	0.5381	0.9016	0.020*	
C27	0.60454 (10)	0.3997 (2)	0.95175 (6)	0.0142 (3)	
H27A	0.5920	0.2755	0.9322	0.017*	
C28	0.93885 (11)	−0.3103 (2)	0.97216 (7)	0.0192 (3)	
H28A	0.9470	−0.3832	0.9358	0.029*	
H28B	0.9004	−0.3899	0.9955	0.029*	
H28C	1.0043	−0.2817	0.9975	0.029*	

### Atomic displacement parameters (Å^2^)

**Table:**

	*U*^11^	*U*^22^	*U*^33^	*U*^12^	*U*^13^	*U*^23^
F1	0.0348 (5)	0.0252 (5)	0.0101 (4)	0.0019 (4)	−0.0027 (4)	0.0002 (3)
F2	0.0312 (5)	0.0224 (5)	0.0208 (5)	0.0136 (4)	0.0040 (4)	0.0047 (4)
F3	0.0358 (5)	0.0264 (5)	0.0141 (4)	−0.0069 (4)	0.0035 (4)	0.0046 (4)
O1	0.0242 (5)	0.0179 (5)	0.0140 (5)	0.0080 (4)	0.0051 (4)	−0.0017 (4)
O2	0.0292 (5)	0.0179 (5)	0.0163 (5)	−0.0003 (4)	0.0084 (4)	0.0024 (4)
C1	0.0160 (6)	0.0160 (6)	0.0153 (7)	0.0009 (5)	0.0023 (5)	0.0005 (5)
C2	0.0176 (6)	0.0156 (6)	0.0163 (7)	0.0012 (5)	−0.0018 (5)	−0.0002 (5)
C3	0.0255 (7)	0.0136 (6)	0.0097 (6)	0.0000 (5)	−0.0006 (5)	0.0004 (5)
C4	0.0237 (7)	0.0185 (7)	0.0139 (7)	0.0007 (5)	0.0066 (5)	0.0000 (5)
C5	0.0163 (6)	0.0182 (6)	0.0156 (7)	−0.0004 (5)	0.0034 (5)	−0.0013 (5)
C6	0.0162 (6)	0.0127 (6)	0.0116 (6)	−0.0003 (5)	0.0009 (5)	−0.0009 (5)
C7	0.0144 (6)	0.0139 (6)	0.0116 (6)	−0.0012 (5)	0.0019 (5)	0.0004 (5)
C8	0.0140 (5)	0.0147 (6)	0.0104 (6)	0.0008 (5)	0.0022 (5)	−0.0009 (5)
C9	0.0121 (5)	0.0120 (6)	0.0130 (6)	−0.0003 (4)	0.0020 (5)	0.0003 (5)
C10	0.0142 (5)	0.0135 (6)	0.0097 (6)	−0.0012 (5)	0.0022 (4)	0.0003 (5)
C11	0.0144 (6)	0.0128 (6)	0.0126 (6)	−0.0002 (5)	0.0041 (5)	−0.0009 (5)
C12	0.0142 (6)	0.0134 (6)	0.0146 (6)	0.0010 (5)	0.0023 (5)	0.0007 (5)
C13	0.0135 (5)	0.0175 (6)	0.0118 (6)	0.0011 (5)	0.0045 (5)	0.0011 (5)
C14	0.0180 (6)	0.0188 (7)	0.0137 (6)	−0.0013 (5)	0.0045 (5)	−0.0007 (5)
C15	0.0165 (6)	0.0185 (7)	0.0142 (7)	0.0013 (5)	0.0033 (5)	0.0008 (5)
C16	0.0150 (6)	0.0197 (7)	0.0121 (6)	0.0020 (5)	0.0022 (5)	−0.0001 (5)
C17	0.0178 (6)	0.0207 (7)	0.0136 (7)	0.0003 (5)	0.0033 (5)	0.0020 (5)
C18	0.0202 (6)	0.0276 (8)	0.0136 (7)	0.0005 (6)	0.0023 (5)	−0.0027 (6)
C19	0.0198 (7)	0.0236 (7)	0.0193 (7)	−0.0006 (6)	0.0021 (5)	−0.0050 (6)
C20	0.0229 (7)	0.0204 (7)	0.0210 (8)	−0.0019 (6)	0.0067 (6)	0.0000 (6)
C21	0.0218 (7)	0.0221 (7)	0.0126 (7)	0.0006 (6)	0.0039 (5)	0.0008 (5)
C22	0.0134 (5)	0.0141 (6)	0.0110 (6)	0.0004 (5)	0.0048 (5)	0.0010 (5)
C23	0.0155 (6)	0.0148 (6)	0.0148 (6)	−0.0003 (5)	0.0050 (5)	0.0005 (5)
C24	0.0205 (6)	0.0134 (6)	0.0160 (7)	0.0013 (5)	0.0080 (5)	0.0003 (5)
C25	0.0198 (6)	0.0175 (6)	0.0142 (7)	0.0065 (5)	0.0071 (5)	0.0050 (5)
C26	0.0163 (6)	0.0203 (7)	0.0122 (6)	0.0026 (5)	0.0030 (5)	0.0025 (5)
C27	0.0156 (6)	0.0156 (6)	0.0119 (6)	0.0005 (5)	0.0039 (5)	−0.0001 (5)
C28	0.0215 (7)	0.0156 (6)	0.0213 (7)	0.0049 (5)	0.0061 (6)	−0.0020 (5)

### Geometric parameters (Å, º)

**Table:**

F1—C3	1.3680 (16)	C14—C15	1.339 (2)
F2—C25	1.3604 (16)	C14—H14A	0.9500
F3—C17	1.3636 (17)	C15—C16	1.464 (2)
O1—C11	1.3681 (16)	C15—H15A	0.9500
O1—C28	1.4304 (17)	C16—C17	1.392 (2)
O2—C13	1.2213 (17)	C16—C21	1.400 (2)
C1—C2	1.389 (2)	C17—C18	1.384 (2)
C1—C6	1.3994 (18)	C18—C19	1.386 (2)
C1—H1A	0.9500	C18—H18A	0.9500
C2—C3	1.377 (2)	C19—C20	1.392 (2)
C2—H2A	0.9500	C19—H19A	0.9500
C3—C4	1.377 (2)	C20—C21	1.384 (2)
C4—C5	1.389 (2)	C20—H20A	0.9500
C4—H4A	0.9500	C21—H21A	0.9500
C5—C6	1.397 (2)	C22—C27	1.3970 (18)
C5—H5A	0.9500	C22—C23	1.4002 (19)
C6—C7	1.4874 (19)	C23—C24	1.3915 (19)
C7—C8	1.3965 (18)	C23—H23A	0.9500
C7—C12	1.3975 (19)	C24—C25	1.377 (2)
C8—C9	1.3976 (19)	C24—H24A	0.9500
C8—H8A	0.9500	C25—C26	1.381 (2)
C9—C10	1.4069 (19)	C26—C27	1.3888 (19)
C9—C22	1.4913 (18)	C26—H26A	0.9500
C10—C11	1.4033 (18)	C27—H27A	0.9500
C10—C13	1.5052 (18)	C28—H28A	0.9800
C11—C12	1.3897 (19)	C28—H28B	0.9800
C12—H12A	0.9500	C28—H28C	0.9800
C13—C14	1.483 (2)		
			
C11—O1—C28	118.02 (11)	C14—C15—H15A	117.6
C2—C1—C6	120.80 (13)	C16—C15—H15A	117.6
C2—C1—H1A	119.6	C17—C16—C21	115.84 (13)
C6—C1—H1A	119.6	C17—C16—C15	120.70 (13)
C3—C2—C1	118.16 (13)	C21—C16—C15	123.46 (13)
C3—C2—H2A	120.9	F3—C17—C18	117.82 (13)
C1—C2—H2A	120.9	F3—C17—C16	118.36 (13)
F1—C3—C2	118.31 (13)	C18—C17—C16	123.82 (14)
F1—C3—C4	118.41 (13)	C17—C18—C19	118.52 (14)
C2—C3—C4	123.28 (13)	C17—C18—H18A	120.7
C3—C4—C5	117.85 (13)	C19—C18—H18A	120.7
C3—C4—H4A	121.1	C18—C19—C20	119.87 (14)
C5—C4—H4A	121.1	C18—C19—H19A	120.1
C4—C5—C6	121.16 (13)	C20—C19—H19A	120.1
C4—C5—H5A	119.4	C21—C20—C19	119.98 (15)
C6—C5—H5A	119.4	C21—C20—H20A	120.0
C5—C6—C1	118.73 (13)	C19—C20—H20A	120.0
C5—C6—C7	121.22 (12)	C20—C21—C16	121.94 (14)
C1—C6—C7	120.01 (12)	C20—C21—H21A	119.0
C8—C7—C12	119.53 (12)	C16—C21—H21A	119.0
C8—C7—C6	120.15 (12)	C27—C22—C23	118.31 (12)
C12—C7—C6	120.26 (12)	C27—C22—C9	122.07 (12)
C7—C8—C9	121.46 (12)	C23—C22—C9	119.60 (12)
C7—C8—H8A	119.3	C24—C23—C22	121.41 (13)
C9—C8—H8A	119.3	C24—C23—H23A	119.3
C8—C9—C10	118.89 (12)	C22—C23—H23A	119.3
C8—C9—C22	118.91 (12)	C25—C24—C23	117.96 (13)
C10—C9—C22	122.19 (12)	C25—C24—H24A	121.0
C11—C10—C9	119.23 (12)	C23—C24—H24A	121.0
C11—C10—C13	117.63 (12)	F2—C25—C24	118.89 (13)
C9—C10—C13	123.06 (12)	F2—C25—C26	118.28 (13)
O1—C11—C12	123.78 (12)	C24—C25—C26	122.82 (13)
O1—C11—C10	114.71 (12)	C25—C26—C27	118.37 (13)
C12—C11—C10	121.40 (12)	C25—C26—H26A	120.8
C11—C12—C7	119.38 (12)	C27—C26—H26A	120.8
C11—C12—H12A	120.3	C26—C27—C22	121.09 (13)
C7—C12—H12A	120.3	C26—C27—H27A	119.5
O2—C13—C14	122.67 (13)	C22—C27—H27A	119.5
O2—C13—C10	120.84 (13)	O1—C28—H28A	109.5
C14—C13—C10	116.49 (12)	O1—C28—H28B	109.5
C15—C14—C13	122.55 (14)	H28A—C28—H28B	109.5
C15—C14—H14A	118.7	O1—C28—H28C	109.5
C13—C14—H14A	118.7	H28A—C28—H28C	109.5
C14—C15—C16	124.73 (14)	H28B—C28—H28C	109.5
			
C6—C1—C2—C3	1.1 (2)	C9—C10—C13—O2	53.22 (19)
C1—C2—C3—F1	−179.81 (12)	C11—C10—C13—C14	56.61 (16)
C1—C2—C3—C4	0.1 (2)	C9—C10—C13—C14	−126.59 (14)
F1—C3—C4—C5	178.50 (12)	O2—C13—C14—C15	10.3 (2)
C2—C3—C4—C5	−1.4 (2)	C10—C13—C14—C15	−169.89 (13)
C3—C4—C5—C6	1.5 (2)	C13—C14—C15—C16	179.83 (13)
C4—C5—C6—C1	−0.4 (2)	C14—C15—C16—C17	175.82 (14)
C4—C5—C6—C7	−177.98 (13)	C14—C15—C16—C21	−3.2 (2)
C2—C1—C6—C5	−1.0 (2)	C21—C16—C17—F3	−179.05 (12)
C2—C1—C6—C7	176.65 (13)	C15—C16—C17—F3	1.8 (2)
C5—C6—C7—C8	47.62 (19)	C21—C16—C17—C18	1.6 (2)
C1—C6—C7—C8	−129.95 (14)	C15—C16—C17—C18	−177.46 (14)
C5—C6—C7—C12	−135.25 (14)	F3—C17—C18—C19	−179.43 (13)
C1—C6—C7—C12	47.18 (19)	C16—C17—C18—C19	−0.1 (2)
C12—C7—C8—C9	−2.8 (2)	C17—C18—C19—C20	−1.2 (2)
C6—C7—C8—C9	174.33 (12)	C18—C19—C20—C21	0.9 (2)
C7—C8—C9—C10	1.9 (2)	C19—C20—C21—C16	0.7 (2)
C7—C8—C9—C22	−178.15 (12)	C17—C16—C21—C20	−1.9 (2)
C8—C9—C10—C11	1.05 (19)	C15—C16—C21—C20	177.15 (13)
C22—C9—C10—C11	−178.94 (12)	C8—C9—C22—C27	−136.61 (14)
C8—C9—C10—C13	−175.70 (12)	C10—C9—C22—C27	43.38 (19)
C22—C9—C10—C13	4.3 (2)	C8—C9—C22—C23	42.13 (18)
C28—O1—C11—C12	15.75 (19)	C10—C9—C22—C23	−137.88 (14)
C28—O1—C11—C10	−167.98 (12)	C27—C22—C23—C24	−1.9 (2)
C9—C10—C11—O1	−179.40 (12)	C9—C22—C23—C24	179.29 (12)
C13—C10—C11—O1	−2.47 (17)	C22—C23—C24—C25	0.8 (2)
C9—C10—C11—C12	−3.0 (2)	C23—C24—C25—F2	−179.19 (12)
C13—C10—C11—C12	173.89 (12)	C23—C24—C25—C26	0.5 (2)
O1—C11—C12—C7	178.11 (12)	F2—C25—C26—C27	179.10 (12)
C10—C11—C12—C7	2.1 (2)	C24—C25—C26—C27	−0.6 (2)
C8—C7—C12—C11	0.8 (2)	C25—C26—C27—C22	−0.6 (2)
C6—C7—C12—C11	−176.31 (12)	C23—C22—C27—C26	1.8 (2)
C11—C10—C13—O2	−123.58 (14)	C9—C22—C27—C26	−179.42 (13)

### Hydrogen-bond geometry (Å, º)

*Cg*1 is the centroid of the C7–C12 ring.

**Table:**

*D*—H···*A*	*D*—H	H···*A*	*D*···*A*	*D*—H···*A*
C4—H4*A*···O2^i^	0.95	2.40	3.3008 (18)	158
C19—H19*A*···F2^ii^	0.95	2.54	3.2326 (18)	130
C24—H24*A*···*Cg*1^iii^	0.95	2.84	3.4579 (15)	124
C28—H28*C*···*Cg*1^iv^	0.98	2.86	3.5461 (16)	128

Symmetry codes: (i) *x*, −*y*+1/2, *z*+1/2; (ii) −*x*+1, *y*−3/2, −*z*+3/2; (iii) *x*, *y*+1, *z*; (iv) −*x*+2, −*y*, −*z*+2.
